# Book Reviews

**Published:** 1823-10

**Authors:** 


### CRITICAL ANALYSIS

OF

### ENGLISH AND FOREIGN LITERATURE

RELATIVE TO THE VARIOUS BRANCHES OF

### Medical Science.

Quæ laudanda forent, et quæ culpanda, vicissim

Illa, prius, cretâ; mox hæc, carbone, *notamus*.—PERSIUS.

---

### DIVISION I.

### ENGLISH.

ART. I.—*A Treatise on the Epidemic Puerperal Fever, as it prevailed in Edinburgh in* 1821-22. *To which is added an Appendix, containing the Essay of the late* Dr. GORDON *on the Puerperal Fever of Aberdeen, in* 1789-90-91-92. By WILLIAM CAMPBELL, M.D. Fellow of the Royal College of Surgeons, one of the Medical Officers of the Royal Public Dispensary, Lecturer on Midwifery, &c.—8vo. pp. 400. Edinburgh and London, 1822.

ART. II.—*A Treatise on the Disease termed Puerperal Fever; illustrated by numerous Cases and Dissections.* By JOHN MACKINTOSH, M.D.—8vo. pp. 323. Cadell, London, 1822.

ART. III.—*Report on Puerperal Fever, in Answer to Queries from the General Board of Health.* By JOHN DOUGLAS, M.D. Licentiate of the King and Queen's College of Physicians.—*(Dublin Hospital Reports,* vol. iii.)

[Continued from page 226.]

WE are much better pleased with Dr. CAMPBELL's performance:* the book is better written; there is sound judgment in many parts of it; and its doctrine seems perfectly orthodox. We wish we could say that Dr. Campbell (tutored by the fatality of the disease,—its protean form,—and its power of resisting every, even the *heroic* remedy, bleeding, the failure of which, in many cases, is abundantly proved by the author's own practice,) had spoken cautiously, diffidently, and modestly of himself, and his success. That he has followed a different course, is no proof of superior fortune; and we are old enough in practice to know that the very best way to prevent readers from placing confidence in the dicta of any medical writer, is to

---

* In order to understand the meaning of the two first introductory lines of our article on Puerperal Fever, in the last Number, it ought to be stated that the works under review then were two only,—those of Drs. MACKINTOSH and CAMPBELL. The title of Dr. DOUGLAS's paper was introduced after the article was written, with the intention of noticing it in the present Number: the lines in question, therefore, do not apply to his performance.

*** We are requested by the Gentleman who favoured us with this article to insert the above explanatory note.—EDITORS.

assume the lofty and untamed language of superior and exclusive triumph over maladies generally deemed uncontrolable.

That Dr. Campbell has allowed himself the use of such language with respect to the treatment and cure of a disease, which least of any permits the adoption of it, we collect not only from the context, but from the very phraseology of his book. In one part of it we are assured, that "the puerperal fever, if detected *early*, and treated upon principle from the commencement, admits of being cured with as much certainty as other diseases, which were at one time considered irremediable:" yet, at page 268, we have a candid and creditable confession, that, notwithstanding the free use of the lancet, and the early detection of the disease, Dr. Campbell and his assistants had been unsuccessful in several cases. In another place we find a charge thrown out, *sans ceremonie*, against the whole of the profession, of using the proper remedies with shameful timidity; as if none had ever bled, and liberally too, in puerperal fever, but Dr. Campbell, his predecessor Dr. Gordon, and his favourite Dr. Armstrong;—as if every, even the most insignificant practitioner in midwifery on this side the Tweed, had not been in the habit of bleeding *ad nauseam*, as well as *ad deliquium*, in the complaint in question, for a long series of years now, as the numerous volumes of the Medical Journals of the day, and the Reports of Hospitals and Dispensaries, fully testify. But the very climax of our author's indiscretion is to be found in his professed desire that all those practitioners who neglect to have recourse to free bleeding in the complaint under consideration, should be held as criminally responsible to the laws of their country for such a neglect! If we are thus to be thrown back to the time of the second Harry of France, when the neglecting to taste the *excreta* of the patients by the physician was punishable at common law,—if the death of a patient, who has not been freely bled during puerperal fever, is to be visited on the head of her medical attendant,—what degree of legal responsibility, do we ask, is even-handed justice to affix to the physician who, in the treatment of seven such patients, spills FIVE HUNDRED AND THIRTY-EIGHT ounces of their blood, and loses them all?*

* The seven cases in question we take at random out of the number of deaths recorded in the book before us.

| | | |
|---|---|---|
| Case I. | 73 ounces of blood, | D.D. |
| Case II. | 90 ounces of blood, | D.D. |
| Case VII. | 110 ounces of blood, | D.D. |
| Case XI. | 82 ounces of blood, | D.D. |
| Case XVII. | 45 ounces of blood, | D.D. |
| Case XXII. | 87 ounces of blood, | D.D. |
| Case XXVII. | 81 ounces of blood, | D.D. |
| Total | 568 ounces of blood, besides what was lost by leeches! | |

3

That we may, however, come more immediately to the purport of the volume before us, we shall conclude the few observations we have felt obliged to make on the spirit of intolerance in which it is written, with wishing that Dr. Campbell had omitted all the critical, desultory, extraneous matter, remarks, episodes, and digressions, which have swelled the book to an unnecessary size; and that, instead of placing Dr. Gordon's unvarnished detail of facts as an appendix to his own work, he had, by a happy conversion, appended the plain narrative of his own practice (which is both respectable and highly to be commended,) to the reprint of the admirable treatise of his predecessor. The world would have lost nothing by such an arrangement; and Dr. Campbell would have gained much in the admiration of those who, strangers to university feuds and lecturers' petty jealousies, can neither applaud the want of modesty in a comparatively junior practitioner, nor encourage him in his attacks on those who have long and successfully toiled in an arduous profession.

Like Dr. Mackintosh, our author has prefixed a sort of literary history of the fever in question; but the two performances are very different, indeed, from each other. Dr. Campbell's history of the disease is remarkable for extensive reading and research, for precision of chronological arrangement, and for happy illustrations or quotations. It is an essay very creditable to the author, and certainly not the least useful part of his treatise. To this eulogium, however, we are bound to append certain qualifications:—First, that a frank statement no where appears in this essay, that every lecturer on midwifery in England, and every medical officer of a lying-in institution, has long been in the habit of considering the disease as only to be subdued by bleeding; and that, therefore, Dr. Campbell's recommendation comes too late for our latitude, although we are glad to see him as an auxiliary in the right cause. Secondly, that many meritorious authors, whose opinions respecting abdominal inflammation in puerperal women, and its treatment, in every way similar to that of Dr. Campbell, have long been before the public,—such as Manning, Ledillot, Park, Chambon, Delaroche, Bigeschi, Granville, Munat, 'Gasc, Gastellier, and others. Thirdly, that Dr. Campbell has given as his own, or, at all events, without any acknowledgment of its being obtained from other writers, information which, in one or two instances, is copied verbatim from other works. We shall only mention one example.

In the enumeration of those authors who differ as to the nature of the disease, Dr. Campbell takes occasion to say, that "Walker, Johnston, Forster, Cruikshanks, Bichat, Pinel, &c. look upon it (the disease,) as inflammation of the peritoneum."

The "Dictionnaire des Sciences Medicales," article *Puerperal Fever*, has precisely the same phrase:—"Walker, Johnston, Foster, Cruikshank, Bichat, Pinel, &c. l'ont considerée comme une affection locale du peritoine." Dr. Campbell has added the names of Gardien, Caparon, Gordon, Armstrong, and Hey, to his list; but, in return, he has omitted that of Gasc, which we find in the French list, and who is in fact a joint author, with Murat, of the article in the Dictionary in question: an article which, from various manifestations in Dr. Campbell's book, we believe that he must have read. Gasc published an *ex professo* essay on Puerperal Fever, in which he considers as *puerperal peritonitis*, and nothing else, some years since; and, as Dr. Campbell, we find, was in France shortly after the appearance of the second edition of that essay, when his attention seems to have been called to this subject, (at page 23, note,) it is somewhat surprising that he should have suppressed Gasc's name altogether. This is not the first time, however, that we have found authors on this side of the Channel dipping freely into that vast ocean of medical knowledge and literature, the Dictionnaire in question, without any acknowledgment. Some few years back, a periodical writer actually copied two-thirds of the matter of an article on the Iliac Passion; in many parts using the same language, and borrowing the same classification, without giving his readers the slightest hint as to the source from whence all his learning and *practical* knowledge proceeded.

*Symptomatology.*—This division of Dr. Campbell's book embraces the characteristic signs by which the disease is known. We were glad to find that the author has not repeated the absurd notion, that *puerperal fever* invariably attacks the patient within the first three days after labour. The present physician-accoucheur at the Westminster General Dispensary had, as far back as 1819, demonstrated the error of such an opinion, and given examples of the disease appearing much later; and we have had occasion, during the present trying season, to see two cases, as severe and as distinct cases of peritonitis post partum as any which Dr. Campbell has brought forward, which came on in the course of the third and fourth week after labour.

We wish it were consistent with the limits of our Journal to give copious extracts of works of merit under review; for we should be tempted to quote the whole of Dr. Campbell's judicious observations in this part of his book. We can recommend them with perfect confidence to the earnest attention of the junior practitioners in midwifery. In the detail of the symptoms of the disease, the superiority of Dr. Campbell over Dr. Mackintosh is really immeasurable. It is a curious coincidence, however, that both of them, in their desire to recommend Dr. Armstrong's treatise on Puerperal Fever, should have, like him, talked of an imaginary and unphysiological variety of the disease, which they have called the *congestive* variety, and of which Dr. Campbell has seen only one case, which he has detailed; founding upon that case (but certainly with becoming diffidence, and not in the positive language of his contemporary, who has completely failed, as we have shown, to prove his acquaintance with the said *species* of puerperal fever,) a description of the variety in question, and of its appropriate treatment.

Before we come to the author's cases, which he has placed immediately after the symptomatology of the disease, we shall discuss, as coming in a more natural order, the points which Dr. Campbell has treated of in a more advanced part of his book, respecting the pathology and seat of the disease, as well as the consideration of the term which ought to be applied to it.

*Onomology of the disease.*—STOTHER is accused of being the author who applied the term "puerperal fever" to the particular complaint which forms the subject of Dr. Campbell's book; but the charge is unsupported by any thing Stother has written. He has stated that puerperal fevers (i. e. fevers attacking women in childbed,) are generally of the inflammatory kind. Surely, such an assertion, correct or not, as it may be, does not imply that the author believed in the existence of any fever exclusively peculiar to lying-in women.

The term "puerperal fever," however, is decidedly bad: it is incorrect—it misleads; yet, after all, if practitioners are convinced, from experience, that such a term is meant to designate an inflammatory complaint of the peritoneum, more or less extensive, (a conclusion which we all seem to have come to long ago,) what signifies the term by which such affection is distinguished? Dr. Campbell himself thinks that, "if we could banish this appellation altogether for the future from medical records, and substitute one which would convey some idea of the real nature of this disorder, it would be advancing a very important step towards the removal of some of the worst prejudices by which it is surrounded," (p. 192;) and he expresses his preference to the denomination given to it by Dr. Gordon, of "abdominal inflammation in the puerperal state." At the same time he acknowledges that such a denomination is vague and indefinite; and, on the whole, he is disposed to adopt the word *peritonitis*. Dr. Campbell is mistaken in supposing that PINEL, of Paris, and Dr. FORSTER, of this country, have been the first to call the disease in question peritonitis. LERY before them, CHOMEL (1728), JOHNSTON (1779), and WALKER (1785,) had pointed out the inflammatory affection of the peritoneum, in the complaint under consideration. Moreover which, it appears, from Pinel's *Nosography*, (edition of 1796,) that he did not then give to the complaint, known under the appellation of puerperal fever, the name of peritonitis, but that of acute enteritis after labour, *(enterite aiguë a la suite des couches.)*

*Etiology*.—Under this head we come to the consideration of what the author has advanced respecting, 1, the *predisposing*, 2, the *exciting causes*. Among the former, Dr. Campbell reckons those various states of the body which render it liable to inflammation. No exciting cause will act unless predisposing causes be present, observes our author. We certainly did not expect to read such a vague and empty phrase as this in a modern book on physic. What else does it mean, or can it mean, than that the school-boy burnt his finger with the flame of a candle, because there was a burning power in the flame, and a susceptibility of being burnt by it in the finger?

If puerperal fever be viewed as an inflammatory disease, again observes Dr. Campbell, it follows that pregnancy must be considered as giving a predisposition to it. (p. 194.) The explanation of this position is contained in the following extract:

"From the moment gestation has taken place, there is a determination of blood towards the uterine system. All the parts, therefore, of which the uterus is composed,—arteries, veins, lymphatics, and nerves, grow; together with muscular fibres and cellular substance. If we examine the peritoneum anatomically, we find that its nerves are considerably larger than in the unimpregnated state: in which, indeed, Haller, Van den Bos, Bichat, and Gordon, have denied their existence. From this increase of the nervous system of the uterus, and those parts connected with it, it is a natural conclusion that the sensibility of these organs will be augmented. Accordingly, the sensibility of the whole system is progressively excited, as may be daily observed from the great peevishness of pregnant females, and from the facility with which they are affected by the most trivial impressions, but more particularly when taken by surprise. The albumen of the blood increases as gestation advances, denoting the presence of augmented irritability. This increase of the abdominal and uterine nerves must powerfully predispose to excitability; of which we have almost daily and convincing proofs, by the ease with which absorption, together with some hysterical affections, such as syncope, palpitations, and many others are produced in some cases. When the term of gestation is completed, and the uterus has thrown off its contents, this viscus contracts itself immediately after to such an extent as to diminish greatly the diameter of the uterine vessels, by which they are prevented admitting the same proportion of blood which they received and circulated during gestation, or before delivery; from which it results that a considerable volume of blood is suddenly diverted from the uterine into the general system. In consequence of these important changes, therefore, it is not only the uterus and parts connected with it which must be considered in a state of congestion, but the whole system will partake more or less of the same condition.

"When we consider, therefore, the plethoric condition of the uterine and general system, and that the blood after gestation must continue surcharged with albumen, we have here causes sufficiently powerful to predispose to inflammation. Of the great sensibility of the system, and its predisposition to febrile affections, in the puerperal state, we have daily and most satisfactory proofs, by the ease with which ephemera, mammary inflammations, and many other affections, are produced, and that from such trivial causes as could scarcely have any influence in a state unconnected with that of child-bed." (p. 195–6.)

Another predisposing cause to inflammation in lying-in patients, is the premature suppression of the lochia. Dr. Campbell, at the same time, admits that the suppression or diminution of the lochia is an effect, and not a cause, of the disease. This must certainly be the case when symptoms of excitement manifest themselves before there is any suppression of the discharge.

The concluding observations of our author on this head are these:—

"In regard to the predisposing causes, I have only further to add, that such parts of the system as are in the greatest state of relaxation have always been remarked to be more prone to inflammatory affections than those whose tone is not reduced. We have here another reason why the abdominal cavity should be more frequently the seat of inflammation than any other part; for it must be allowed that the abdomen is in a state of debility after parturition, in consequence of the distention of its parietes, and the pressure upon its contained organs, during gestation; together with the violent action of the parts at the time of delivery." (p. 201.)

The exciting causes of puerperal peritonitis (or fever), we are told, are as numerous and diversified as in any other disease of an inflammatory nature.

"Among the exciting causes which have been usually remarked as possessing influence in producing the disease in question, may be mentioned—applying the binder round the abdomen with unusual firmness after parturition; the sudden and forcible detachment of the placentary mass, and retention of the secundines; injuries before and during the process of parturition, by brutal violence, or the use of instruments, and consequently severe labour; mental emotions; exposure to cold; premature use of stimuli, whether food or drink; impure diet; lactiform metastasis; infection, and a noxious constitution of the atmosphere." (p. 202.)

Dr. Campbell enters at some length into the consideration of the three latter, as they have been supposed by different persons to have a considerable share in producing puerperal fever. With regard to the others, he merely offers some general remarks, which it is the least to say that they are pertinent and judicious.

Dr. C. is friendly to moderate compression of the abdomen after labour, by means of a bandage applied with a certain degree of firmness. He differs from DENMAN, who recommends its application to be delayed for six days after parturition.

"With regard to the management of the placenta, it is agreed upon by all accoucheurs that the extraction of the mass ought never, except in cases attended with uterine effusions, to be conducted with precipitation or force, from such proceedings having been too frequently attended with formidable consequences, more particularly hemorrhage. But, although we are not generally to accelerate the extraction of the mass, I must cordially agree with our distinguished professor in thinking that its retention longer than an hour would be injudicious, and ought not to be permitted: for, by the end of this time, the passages will contract so much as to render the introduction of the hand, with a view to withdraw the mass, difficult; and, from the exertions required and consequent irritation, we may occasion the very accident which we wish to prevent. But, whatever time the extraction may require, no pretence can justify a practitioner in leaving his patient until he has accomplished it." (p. 203—4.)

Our author justly boasts of knowing nothing of his own experience respecting that species of fever which results from the retention and putrefaction of a part, or the whole, of the placentary mass. If, instead of a number of well-instructed pupils acting under his direction, Dr. Campbell had to attend to the blunders of sundry ignorant midwives, with many of whom it is a standing joke that they *pull a strong pull* at the cord, he might perchance have had more than one occasion of witnessing the *fever* which follows the retention and putrefaction of a portion of the placenta. He would not, then, have hazarded *his supposition* and opinion that this *fever* is identically the same with the "noted puerperal fever." Never did a physician hazard more unluckily an opinion upon hearsay or written evidence, than Dr. Campbell has on the present occasion. We can tell him from experience, derived from sources to which we have just hinted, that no two fevers differ more widely from each other in their characters, as well as in the treatment they require, than this identical fever from putrefied animal matter absorbed into the system, and the puerperal fever of Dr. Campbell, if he means puerperal peritonitis. In the first place, it never shows itself before the fifth or sixth day, as the earliest period. In the second place, it does not begin with rigors nor head-ache; both these symptoms appearing at a more advanced stage of the disease. In the third place, we have prostration of strength, evening exacerbations of febrile symptoms, perspirations, &c. for two, three, and four days before any pain manifests itself in the abdomen; and, when this does show itself, it is neither so acute nor so permanent as in peritonitis. We might go on with this parallel to a greater length, but we have no room for more than this other important observation:—that were we, on being called to a case of fever, such as we have described, and assuming Dr. Campbell's view of its identity with puerperal fever, to bleed the patient, the result would be the speedy extinction of life. Purgatives,—the mineral and vegetable acids, but, above all, the nitric acid,—bark, &c.: these are the *auxilia* for us. Dr. Campbell has shown too much sense, throughout his work, to scorn receiving a little advice from fellow practitioners.

We pass over the many apt remarks of our author on the remaining exciting causes, as we trust our readers will consult the work itself, and come to the subject of infection.*

---

ART. VI.—*An Exposition of the Principles of Pathology, and of the Treatment of Diseases.* By DANIEL PRING, M.D. Member of the Royal College of Surgeons, London.—8vo. pp. 512. Underwoods, London, 1823.

NOTHING, certainly, can be less consistent with the notions we should *a priori* have formed of the time and circumstances an author would select for the composition of an abstract argumentative discussion of pathological doctrines, than those under which the work before us was written. Instead of finding Dr. PRING quietly seated in his closet, shut out from the world, and with the ponderous tomes of "skilful chirurgeons and more learned physicians" of former times piled before him, we are informed that his "Exposition" was begun and ended during a tour which occupied some of the summer months, and that his books of reference consisted in an odd volume or two of Shakspeare and Don Quixote. This explanation the author has deemed it proper to make, as serving to account for the references made to the writings of others being so few; and even these it appears have been subsequently added.

The work commences with a minute and critical review of some of the most important pathological doctrines, or systems, which have at different times been in possession of the medical schools; each of which shared nearly the same fate, having for a time been regarded as the greatest discovery of the age, which was to be handed down to posterity as a monument of the wisdom and learning of their fathers. Since, however, none of these have been received with the veneration that was anticipated,—and since, in truth, they have all been found incorrect or deficient,—it may possibly be inquired, what profit is likely to result from renewing their discussion? In answer, we should

* We are obliged, by the pressure of other matter, to postpone the remainder of this article until the ensuing Number.

reply, first, that such an inquiry is calculated to inspire a certain degree of caution in admitting, with the readiness that is so often done, the novelty of the day as the true solution of all the arcana in physic; and, secondly, we shall find that, although generally carried too far, and often inaptly applied to the explanation of the phenomena of disease, there are nevertheless many views to be found among the exploded doctrines, which were both founded on observation, and are capable of affording important therapeutical indications.

Among these doctrines, the *humoral pathology* is the one of earliest date, which continues to exert any influence in medical reasoning: indeed, we are inclined to think that this doctrine is less in disrepute now than in the days of Cullen, and that it still mingles in our theoretical and practical opinions to a greater extent than medical men are aware of, or perhaps willing to acknowledge. It is, as our author justly remarks, the popular system; and even professional men are often fain to apply it to the elucidation of some morbid phenomena, rather than avow their inability to explain them at all. "Thus they oftentimes gain credit for superior knowledge; and those who can condescend to accept reputation upon such terms are seldom deficient in the information that an appeal to the fallacies and prejudices of human nature is generally more successful than an address to its better understanding."

Although the mechanical part of this doctrine be now exploded, still the leading principle, which attributes diseases to something wrong (or, to use the technical language, "peccant") in the condition of the fluids, is by no means so completely discarded. Indeed, to a limited extent, the pathology of peccant humors is extremely plausible: for example, certain products of disease are capable of giving rise, in another individual, to the same disease from which they originated,—as small-pox; and it is to be observed that, in this instance, the plausibility is increased by the quantity of matter which is generated and evacuated by the skin. Certain diseases of internal parts were observed to cease on the occurrence of cutaneous eruptions; and other evacuations, as the urine, sweat, &c. are to this day regarded as critical of disease. So far we have little to find fault with; but the humoralists went further, and, applying the principles of hydraulics to the animal body, endeavoured to resolve morbid phenomena into mechanical movements. The fallacy of these opinions is thus pointed out by our author:

"We will suppose there to exist in the system a peccant humour, or morbific matter: in what mechanical way can it produce the phenomena of pneumonia, or of synochus? We will say that this fluid produces disease by its action, for the sake of simplicity, upon the heart: what is the *modus operandi* by which the configuration of the particles of a fluid can, by a mechanical agency, make the heart beat 130 instead of 70 strokes in a minute? In order to produce such a change in the action of the heart, the fluid must have some relation with its *moving powers:* if these powers are influenced mechanically by such a fluid, they must themselves be of a mechanical kind; but, as the known mechanical powers of motion are such as arise either from springs or weights, and as the existence of such powers cannot be recognised in the construction of the organ in question, it is necessary to look for other causes of an effect, with which those which have been assigned have no sensible or analogical connexion. If these causes, then, of the action of the heart are not of a mechanical kind, it is necessary, as their existence is inferred from their effect, to distinguish them by some other denomination.

"By whatever term the causes of the action of the heart may be expressed, if they are said to be not mechanical, then our peccant humour, or morbific matter, (if, indeed, it is at all interested in the effect,) produces the change from 70 to 130 beats in a minute, not by a mechanical mode, but by a relation with actuating properties which are confessedly not mechanical. Hence, conceding the existence of such a morbific matter, if its mechanical relation can be only with a mechanism, which does not exist in the present case, its inadequacy to explain the phenomena imputed to it in this instance is sufficiently obvious; and its fallacy is demonstrated by the proofs of a dependence of these phenomena upon powers which, having no analogy with those of mechanism, must be otherwise designated."

But it is no less obvious that the phenomena in question cannot be explained on any principle of chemistry, so that we are driven to a third set of properties called *vital.* The author here enters into some discussion with regard to the nature and action of these same vital properties, in which we recognise the same ingenious, but abstract, method of reasoning which characterised his former work.* This we pass over, being unable to form any other than a negative idea of a vital property,—viz. something producing phenomena in the animal body, which cannot be explained on chemical or mechanical principles.

Granting that diseased actions cannot be explained on any mechanical operation of the particles of the fluids, still a certain relation between such particles and the size of the vessels through which they are to circulate, is obviously required for a due performance of the functions: when, however, such relation does not hold good, it is supposed to give rise to various derangements. Thus we hear every day of vessels being infarcted with blood, the gall-ducts blocked up with vitiated bile, &c. The author, however, argues against the truth of these occurrences taking place to such an extent as to give rise to the

* Pring's *Indications, &c.*

*error loci* of the ancients, although he allows that this last borders closely upon *congestion*, the fashionable error committed by the circulating fluids at present. That the blood varies in its consistence and sensible qualities at different times, is well ascertained, although it is not easy to trace the effect of such changes on the circulating system. If the blood be thicker than usual, it must require a greater degree of force in the action of the heart to give the same velocity to an equal bulk; and, consequently, either the heart must act with greater vigour, or the circulation become slower; and Dr. Pring conjectures that the additional weight possessed by the blood, in the case supposed, may have some effect in augmenting the injury to the brain, where determination to that organ occurs. In considering the opposite state of the blood, the author makes the following remarks:

"The same view of a mechanical relation between the blood and the heart must obtain, when this fluid is preternaturally thin, as under the condition of it which we have just considered; that it is the opposition of a weight to an active power. The proportion of serum to crassamentum is sometimes more than may be thought natural, in persons who are in tolerable health. But we are chiefly to consider what effects may arise from this thin state of the blood, in those extreme cases in which, from previous losses of this fluid, the crassamentum bears a proportion to the serum, as perhaps one to five. We observe, in these cases, that the active and resisting powers are rarely balanced: the heart retains its power of action, and operates upon a diminished weight; there is seldom a slow pulse in those who have lost much blood, either by the lancet, by an accidental wound of an artery, or by uterine hemorrhage. On the contrary, the pulse is apparently full, bounding, and ranging perhaps from 90 to 120; and, if under the latter rate, is easily raised up to it by the most trifling excitement. I know not in what way this accelerated motion of the heart, and apparently vigorous circulation, after immense losses of blood, are to be accounted for, but by supposing that the power of the heart, its irritability, or however else denominated, is not diminished by such loss of blood; or, if diminished, that it is not in a ratio to the reduction of weight against which it acts.

"As we have remarked vertigo and alienation of mind to have been connected with a condition of the blood in which the crassamentum was preternaturally abundant, so it also happens that the head is most commonly affected in this reverse state of the blood, though the symptoms are not precisely the same. It is commonly (and in my own experience it has been, I believe, invariably,) the case, that those who have sustained great losses of blood suffer more or less from what is called determination to the head. The symptoms most commonly are intense pain, and throbbing in the forehead or back part of the head, with a pulse seldom under ninety. I have known these symptoms to proceed on, with a pulse of from 120 to 140, to delirium, serous apoplexy, and death. As this has occurred in a case where the loss of blood was not connected with any previous disease, so the determination, if it may be so called, in this instance, could be fairly imputed to no other cause than the loss of blood." (p. 22, 23.)

It does not appear, however, that either the thickness or tenuity of the blood exerts any considerable or marked influence on the phenomena of disease; of which, indeed, these conditions are to be regarded as the effects rather than the causes.

*Doctrine of Spasm.*—It was chiefly through the opposition of CULLEN that the humoral pathology was driven from the schools, and this doctrine substituted in its place. The best illustration of this view is derived from the phenomena of fever, which were supposed to consist essentially in spasm of the extreme vessels. The effect of such spasm was the suspension of the particular discharge, which, being unable any longer to pass through the minute vessels in this unnatural condition, was of necessity forced into other channels, giving rise to the various symptoms constituting fever. The suppression of the perspiration, it is obvious, can only operate in one of two ways,—viz. by determinating a larger proportion of fluids to other parts, or else by preventing the evacuation of something noxious; and, in either case, the explanation is founded on something besides the spasm: the former is mechanical, and the latter is an acknowledgment of the existence of "peccant humours." Besides these two alternatives, our author proposes the following:

"But the production of the phenomena which have been ascribed to checked perspiration, is liable to the alternatives of two modes; perhaps a third might be added. If the entrance of fluids into the extensive system of cutaneous vessels is resisted by a preternatural contraction of these vessels, it is obvious that those vessels which are not cutaneous must contain more than their natural proportion of fluids: this state would be one of general internal plethora. But we observe that, as a consequence of suppressed perspiration, one organ always suffers principally; that there is a particular determination to one seat. If a contracted state of the cutaneous vessels could throw an unnatural proportion of fluids into the internal ones, so a preternatural dilatation of the vessels of any other seat or system would deprive the cutaneous vessels of their natural proportion. We have then to choose whether disease is maintained by suppressed perspiration, or whether the suppression of perspiration is a consequence of disease.

"If we consider here merely the order of occurrence, it would appear that the disease—say, for example, acute rheumatism,—was produced by suppressed perspiration. At the same time, there is no reason why the muscles, rather than any other parts, should suffer from the suppression of perspiration; seeing that the simple circumstance of a diminished circulation in the skin is equally relative to the vascular system of every seat. In order that one seat should suffer a vicarious determination rather than another, it is necessary to suppose a predisposition in such seat which does not exist in others. This predisposition is very nearly allied to disease; and the admission of it goes near to confessing that the circumstance of a suppressed perspiration was preceded by processes of disease in that seat which afterwards exhibits its chief phenomena.

"From this view of the subject it would appear, that the diseases which are imputed to a contraction of the skin by cold precede the circumstance to which their origin is attributed; that the operation of cold is to determine the occurrence of symptoms which would be developed by spontaneous processes of disease which had already commenced. It is, however, probable that, if the processes had been entirely spontaneous, the form of the disease might have been modified by running into another stage of change before symptoms were declared. A woman who is menstruating may at one time get wet in the feet, or elsewhere; and, though the exposure to wet and cold may at this time be for a considerable period, she may in consequence suffer no inconvenience. At another time, when the same cause operates in a less degree, fever might speedily occur, with a local inflammation of the lungs. The disposition to this disease of the lungs must have preceded the exposure to cold, and the disease in the lungs will run perhaps a protracted course,—may terminate in phthisis,—and may substitute the discharge from the uterus, the vessels of which, but for this vicarious determination, might have recovered a disposition, which was only transiently suspended, to pour out their natural periodical secretion." (p. 35—37.)

If, however, we allow that predisposition to disease exists in the organ prior to the exposure to cold, it is impossible to admit spasm of the cutaneous vessels as the first link in the chain; and that such predisposition does exist, seems abundantly evident from a variety of facts. Exposure to cold produces very different effects at different times, and in different individuals: in one it gives rise to catarrh, in another to rheumatism, in a third to diarrhœa, and in another no diseased action of any kind occurs. Now, the fact of the mucous membrane of the fauces being affected in preference to that of the bowels, shows that part to be in a condition differing in some way or other from that of the former; and it will obviously be difficult to distinguish between this and actual disease: at all events, it exists anterior to the application of the cause which excites spasm of the minute vessels. The truth of this is further proved by the circumstances of the decreased action continuing after the constriction or spasm has been removed, as evinced by free perspiration; or, supposing the suppression of the cuticular discharge to have given rise to a state of congestion in some other part, by determining an increased quantity of fluid into vessels unaccustomed to receive it, yet this effect must be at once removed by the first blood-letting, although the disease frequently remains unabated. In short, in the words of our learned author,

"Although it cannot be denied that the cutaneous vessels may be constricted by cold, and in this way produce some change in the balance of the circulation, yet such a change is totally inadequate to explain either the nature or duration of the disease which might succeed to it. It is necessary, for the origin of such disease, that its processes should have commenced before the occurrence of the alleged cause; and its continuance, together with all the irregularities and changes which mark its course, are dependent, not upon a condition of cutaneous vessels, which is temporary, but upon the disposition of its seat; and this disposition is the result of modified relations of the properties of life, both among themselves and in regard to their mechanical and hydraulic connexions." (p. 39.)

The application made of the doctrine of spasm to the phenomena of inflammation, is next examined at some length, and its entire insufficiency for their explanation is fully pointed out: but into this part of the subject we conceive it unnecessary to enter.

*Doctrine of* Brown.—While Cullen yet taught the doctrine of spasm from the first medical chair in Europe, the rival system of Brown was promulgated at the same school, and gained many proselytes, who were probably attracted by its alluring, but deceitful, simplicity. The doctrines of Brown never, however, obtained any considerable influence over the practice of medicine in this country, and soon ceased to be regarded in any other light than as a brilliant production of genius, uncorrected by judgment and at variance with fact. Yet its reputation, however transient among his countrymen, still ranks so high in the south of Europe as to constitute, with some modifications, the *nuova dottrina* of the Italian physicians. On this account, we shall enter somewhat more fully into the subject than we should otherwise have deemed necessary, and beg to call to the minds of our readers the leading principles of this system in as few words as possible. Brown held that there existed in animals a capacity for the phenomena of life, called *excitability;* to call which into action, the application of certain influences was necessary, and these were called *stimuli.* Upon the relative state of these depended all the varieties of health and disease. When just a sufficient quantum of excitability was consumed by the stimuli, the individual enjoyed natural health: when the stimuli were more abundant, but not to such excess as to exhaust the excitability, a preternatural vigour was the consequence, and this was called the *sthenic* diathesis;—when the stimuli were still more abundant, or the excitability rather deficient, so as to become exhausted, this constituted *indirect debility;*—when the stimuli were not sufficient, the excitability was supposed to accumulate, giving rise to a state of *direct debility.* The indications of cure were, of course, quite clear. The sthenic state was to be removed by diminishing the stimuli; and the indirect debility by a similar method, more gradually adopted, so as to give the excitability an opportunity of accumulating. The direct debility required an increase of stimuli. Thus was the whole system of pathology and therapeutics reduced to a degree of simplicity unattained by any previous author, and having no other drawback except that of being utterly inconsistent with the phenomena of disease, and leading to methods of treatment which were demonstrated by daily experience to be not merely inefficient, but positively and eminently pernicious.

The deficiencies of Brown's doctrine are extremely well pointed out in the work before us. It is stated that animals possess a principle called excitability, which is constantly going on suffering daily exhaustion from the cradle to the grave, without any apparent source of supply;—we are told that it accumulates at one time, and at another recovers itself, if exhausted by the too free application of stimuli; both of which circumstances would of necessity imply some means of its regeneration.

All diseases, according to the Brunonian system, come under one of two descriptions,—viz. increase or diminution of strength; but, unfortunately, no criterion is established by which to judge of the degree actually possessed by the subject of any disease.

"If the strength of a subject is judged of by his muscular power, we find that this may be the least when the sthenic diathesis prevails; as, in forty-eight hours after the commencement of an inflammatory disease, the subject who could before walk perhaps thirty miles in a day, can scarcely walk across the room. Depletion, according to the doctrine, is the remedy for this state of disease; the effect of depletion is to produce debility, yet the debility in the animal system is already excessive: the animal system, then, does not appear to furnish the criterion of strength or weakness.

"In the organic system, it would be difficult to find any one state, or definable collection of symptoms, which is not common both to the sthenic and asthenic classes of disease. The rate of the pulse may be the same in a robust man, in the commencement of pneumonia, as in one in the last stage of consumption, who has suffered a chronic course of depletion and abstinence, and from whom the means of strength have been for perhaps many months withheld. It is equally obvious that no other state of the pulse can be assigned, which is not common to diseases whose history and character are totally different. The same remark is true, if the criterion is looked for in any other organ or function: thus, the appetite may be the same in health and under the diseases of cancer or consumption; the urine may be as copious with a schirrhous liver as if the liver were not diseased, or as scanty in dropsy as in typhous fever; or, in some forms and stages of these diseases, neither more copious nor scanty than in a state of health. Perspiration also may be abundant both in health and disease; and the tongue more furred every morning in those who get up to go about their daily labour, than in a person who is suffering under chronic rheumatism, with a pulse of 110 and buffed blood." (p. 64—65.)

The author goes on to show that, as strength may be supposed to depend upon the due performance of the various functions, such as takes place during health, so debility might be at first sight regarded as a general consequence of any deviation from this state. But a more close examination will show that vital energy is not, during health, so great, but that the functions may be performed with greater energy; as the action of the heart is increased in fever, &c. showing that the greatest functional energy is not necessarily the consequence of health. This leads to some inquiry with respect to the "power with which high functions are exercised." Suppose the heart to beat at one time seventy strokes in a minute, and at another to beat one hundred, we infer that the degree of power or excitement is greater in the latter than in the former case. The author here takes occasion to enter into a long discussion on the difference between degree and quantity, their nature and relations: to this disquisition we must refer any of our readers who may be disposed to follow minute and abstruse reasoning, without any very obvious application to the subject in question,—or, at least, without any such that we are able to discover. And here we would crave permission to hint to Dr. Pring, that medicine and metaphysics are branches of science so entirely opposed, as to create inevitable confusion when it is attempted to unite them in one treatise. It is this passion for abstract illustrations,—for general principles,—and, we must be permitted to say, for metaphysical refinement, which prevented the "Indications" from ever obtaining any considerable share of public attention; much less such share as the mass of acute reasoning and sound principles contained in that work justly claim for it. The same causes, we fear, will operate in preventing the one before us from meeting the notice it deserves. If Dr. Pring would write one book on medicine without metaphysics, and another on metaphysics without medicine, we conscientiously think he might take a high station in either department.—But to return.

The principal morbid phenomena which have appeared to reflect credit on the Brunonian doctrine, are those resulting from exposure to cold. It is universally known that the sudden application of heat, under such circumstances, is attended with the most pernicious consequences; and the explanation, in conformity with this doctrine, is, that the sedative effect of the cold, in the absence of the stimulus of heat, has permitted the excitability to accumulate; which excitability, if too quickly and powerfully called into action, may be attended with effects too violent for the powers of life to resist.

But, although we reject the pathology of Brown, and his explanation of these phenomena, on the principle of accumulated excitability, yet there is one fact in accordance with his views, and perhaps in some degree originating in his system, which is of the highest practical importance: we mean the absolute necessity of supplying stimuli, in cases of debility, gradually and with caution. It must have occurred frequently to all practitioners, (particularly to those connected with institutions, which lead them to the bedsides of the poor,) to witness the bad effects resulting from the improper administration of too stimulating diet to persons convalescent from fever. One of the most common effects of this stimulation is increased determination to the head, frequently threatening apoplectic seizure. On this subject we request the attention of our readers to the following remarks of Dr. Pring, which, we believe, are borne out by the fullest experience.

"It is common, in these severe and threatening forms of cerebral disorder, notwithstanding previous loss of blood, to resort to the lancet, and to repeat the bleeding, copiously and frequently, if the symptoms continue. So far as I have had opportunities of observation, this practice, carried to any great extent, to the neglect perhaps of other measures, has generally had a fatal termination. I have been in the habit of confiding in purgatives, to the almost total neglect of the lancet; but these purgatives have not been of a milk-and-water kind. If, after such copious loss of blood, the pulse has suddenly got up from eighty to one hundred and thirty or forty in a minute, with intense pain in the head, perhaps slight incoherence of speech, restlessness, &c. I have given six grains of calomel, and the same quantity of James' powder, followed by a two-ounce draught of salts and senna, with half a dram of jalap in it. If this has been rejected, it has been repeated in less than an hour, and repeated as often as it was rejected, until it has produced copious evacuations from the bowels. One, and perhaps an important, effect of such purgatives is to make a great revulsion to the whole intestinal canal, which is commonly followed by almost perfect relief of the head, and perhaps an immediate subsidence of the pulse to 100, or 110. The tendency to disorder of the head is afterwards easily kept within safe bounds, by small, but repeated, doses of Epsom salts; or an exacerbation, if it occurs, may be reduced by a repetition of the former purgatives. If, notwithstanding a full and continued purging, the disorder should persevere in its course, I have reason to think, from the results of cases essentially analogous, that putting the system suddenly under the full influence of mercury is more likely to subvert the disposition to cerebral disorder, than the employment of the lancet. But, before this measure is resorted to, the fatal termination of the case (unless a vigorous effort is made to avert it,) must be so decidedly indicated, that the further prosecution of curative endeavours seems little better than a forlorn hope. In a pretty considerable experience, I have never met with a fatal instance of disorder of the head of this kind when, however threatening and severe the symptoms might be, the plan of treatment by purgatives, nauseating, and perhaps emetic remedies, has been wholly or chiefly depended upon. And, so far as my recollection serves me, all the fatal cases which have come to my knowledge have been those in which the treatment of disorder of the head of this kind has been rested wholly or principally upon the use of the lancet." (p. 85–6.)

The author next favours us with some valuable remarks on the treatment of *fever;* the general result of which is the recommendation of free and continued purging, and avoiding the repetition of blood-letting. As an example: a man, between forty and fifty years of age, was attacked with the usual symptoms of violent fever, in which the head was considerably affected, and the pulse within eight or ten hours had amounted to 136 in the minute. He was bled to the extent of twenty ounces, took eight grains of calomel, with a like quantity of James's powder, followed up in an hour by a black dose, containing half a dram of jalap; and, after the operation of the purgative, he had a grain of tartar emetic every six hours. In twelve hours from the first purgative, he had three grains of calomel and six of James's powder; followed, in twelve hours, by a black draught, containing a scruple of jalap. In about thirty hours the pulse had come down to 105; and the bleeding was not repeated. "By a treatment with a similar design, (says Dr. Pring,) I have brought a pulse in fever down, in twenty-four hours, from 120 to 56 in a minute, without bleeding." (p. 90.) At page 95, the inexpediency of repeated bleedings is again urged, and small doses of tartar emetic, or large ones of squills and nitrate of potass, recommended as more effectual in bringing down the pulse. At page 98, we have the following remarks upon this subject:

"On the propriety of blood-letting in typhus, I have reason to think it may be beneficial in the commencement, and the repetition of it topically, or perhaps generally, in small quantities, may be advantageous, if judiciously employed in the course of the disease. I once attended three patients in the same house, who had at the same time the inflammatory form of typhus: their circumstances, their symptoms, and the degree of them, were about the same; the pulse in each was not under 130 within the first twenty hours after the rigors, a rate which indicates a dangerous accession of fever. I have reason to doubt whether a patient would recover under any treatment, whose pulse amounted to 140 in a minute within the first twelve or fourteen hours after the rigor. If the patient does recover, the event will do great credit to the treatment, and the parties interested will also be something indebted to their good fortune. I bled only one of the three patients just alluded to, and trusted to purgatives, &c. with the rest: they all recovered, but the disease ran a more mitigated course in the one that was bled than in the other two.

"A considerable loss of blood, whether by two or three large bleedings, or by many small ones, has appeared to be prejudicial in typhus: the cases in which this practice has been carried to a great extent, are among the fatal ones which have come to my knowledge, and death has been said to have happened from serous effusion into the ventricles of the brain. I would not be understood to insinuate the condemnation of blood-letting in typhus: a practitioner must be free to employ any measures that are indicated, and he should by no means limit his resources, for the sake of consistency, by a systematic opposition to any one remedy."

Emetics are not favourably spoken of by our author, who has seldom given them except in the form of nauseating doses: he has known an emetic, in the commencement of a fever, followed by an apoplectic seizure before its operation was over; an occurrence which the patient did not survive many hours. We suspect, however, that this case has made a greater impression on the author than the circumstance appears to warrant; for this effect (if indeed it was an effect of the emetic,) is so extremely rare, as scarcely to admit of our taking it into the account; while, so far as our own experience goes, no remedy so frequently cuts short the course of a fever as the one in question.

It sometimes happens that, in spite of every means employed, typhus runs on unchecked, terminating in a state of coma, which, for the most part, is the forerunner of death: in such cases, Dr. Pring recommends the rapid introduction of mercury into the system, so as to bring the patient speedily under its influence. This is done by giving ten grains of calomel every six or eight hours; and he has known all the alarming symptoms cease so soon as salivation became established. The use of mercury, however, is by no means limited to this stage of the disorder, but it may be employed in arresting the tendency to a fatal termination, "when, from the character or degree of the symptoms, the more common remedies appear inadequate to this end." (p. 102.) How entirely the whole of this method of treatment differs from the stimulating system of the Brunonians, it is unnecessary to remark.

*Pathology of the Determination of Blood.*—This is one of the most fashionable among the pathological doctrines of the day; the phrase *determination* at once expressing the fact of an unusual accumulation of blood in the part, and the manner of its occurrence. This doctrine owes much of its respectability to the support of the acute and philosophic Dr. PARRY, whose attention seems to have been first directed to the subject from observing the effect produced by pressure on the carotids, in a case of hysteria; the symptoms being controlled by this interruption in the flow of blood to the brain. The different circumstances which may give rise to accumulation of blood in an organ, are resolved by our author into three: 1st, mechanical obstruction; 2d, accelerated action; and 3, preternatural dilatation of vessels. Mechanical obstructions may operate either upon the arteries or veins: thus, a tumor pressing upon an artery so as to diminish the flow of blood through it, a large portion must, of necessity, be sent to the parts above this interruption; or the gravid uterus, by compressing the iliac veins, may prevent the return of blood from the lower extremities, and so give rise to accumulation of blood in these parts:—this, however, scarcely comes under the definition of determination, but would be better expressed by *retention* of blood. Those who believe in the existence of spasm will find in it another cause of mechanical obstruction. The second method of explaining local determination is obviously dependent upon the arteries themselves, and consists in allowing them an independent power of action. Whether arteries do really possess a power of alternate contraction and dilatation, does not admit of such easy solution as might at first sight be imagined; and, although the various arguments are discussed with much ingenuity and at some length by Dr. Pring, we shall confine ourselves to a very brief statement of them. When an artery is laid bare, it is seen to be a cylinder of fixed dimensions, without any alternate contraction and dilatation: if, however, the finger be applied, it is felt to pulsate, which led Dr. Parry to conjecture that the degree of pressure exerted by the covering of arteries was the cause of their pulsation. If, instead of exerting pressure with the finger, it be "lightly moved or rolled transversely" over the artery, it will be found to present a uniform distention. This experiment has been habitually practised by our author, and he regards it, when taken in conjunction with the appearance presented by a denuded artery, as decisive against their possessing a power of alternate contraction and dilatation. Another argument in favour of the independent action of arteries was deduced from some supposed phenomena of disease: thus, it was confidently stated by various writers, and among others by Dr. Pring, that the vessels in an inflamed part,—for example, in a whitlow,—pulsated more frequently than the branches leading to it. It appears, however, that our author has been led, by more extended observation, to doubt the accuracy of his former opinions. The strokes of an artery in two different parts, it must be observed, are not synchronous, but that of the artery next the heart is anterior to that of one at a greater distance: this circumstance rendering it impossible to count the pulsations in two parts at the same time with any satisfactory degree of accuracy; and, consequently, the observations of Dr. Pring and others on this point do not apply to the comparative frequency of the pulsations in different arteries, or different portions of the same artery, at the same time, but at successive times. Now, it is quite obvious that an examination, first, of the pulsations in one artery, and then in another, gives no satisfactory result with regard to their relative action; as the heart may, and frequently does, vary in its pulsations, in a manner quite sufficient to account for any difference that may be observed. The most simple method of performing this examination would be for two persons to feel the different arteries at the same time; and we are informed by the author that, in the only instance in which he practised this, the pulsations in the inflamed and healthy seat were numerically the same.

"Although I incline to the opinion that the proofs are stronger against than in favour of the alternate dilatation and contraction of arteries, yet it must be allowed, in the first place, that all these proofs are not uniform in their occurrence; and, in the second, that there are circumstances with which the supposed absence of this faculty is at least in analogical disagreement: such as the facts, 1st, that an artery will empty itself of its blood beyond a ligature; 2d, that there is more blood, or, as is conceded, a greater velocity of blood, (which is much the same thing,) in a given space of an artery at one time than at another, which effect would ensue merely from the blood being thrown into the arteries by the heart, in successive quantities; 3d, the irregular manner in which blood is projected from a wounded artery; 4th, that no visible pulsation of a denuded artery is produced by a ligature, whether the vessel is partially or completely constricted by it, which one would expect to be the case, if the pulse were given, as explained by Dr. Parry, by a circulation locally impeded, and a consequent effort on the part of the blood to restore the calibre of the artery in the situation of such impediment. To these circumstances must be added the irregularities or disagreement of the pulse of different arteries, which are certainly sometimes observable, if the mode of examination is in any degree to be trusted, under inflammatory disease. These facts collectively will leave still, I fear, some shade of doubt on a point of physiology which is both interesting and important. It furnishes no boast of modern researches in physiology, that, although the circulation of the blood has been discovered nearly two hundred years, the manner in which this circulation is accomplished is not only not ascertained, but, as a matter of opinion, is one on which physiologists are not agreed." (p. 117—119.)

The mechanical powers of the circulating system are obviously insufficient to account for the phenomena, without the intervention of some other agency. 1st. The vital properties of the vessels may produce some influence on the blood, so as to facilitate its transmission; or, 2dly, this may be effected by the capillaries. The fluidity which is preserved by the blood in arteries would seem to show the existence of the former, as it is difficult to account for this phenomena, without admitting the intervention of that mysterious agency which is called vital; but, granting this to be the case, the fluidity of the blood can only operate indirectly in facilitating the circulation.

The operation of the capillary system as an assistant to the heart in effecting the circulation, is probably more important. The secerning extremities of arteries form such an extensive aggregate of terminations to these vessels, and their functions are effected by means so independent of, and frequently in such direct opposition to, all the laws of hydraulics, that we cannot but acknowledge, as well the magnitude of their operation, as the peculiarity of the means by which such operation is effected. "By the agency of these properties, (says our author, p. 127,) we see the vessels of this system making their peculiar separations; we find the minutest order of tubes separating the thickest fluids, and rejecting the thinnest; we see those of a larger order conveying thinner fluids, and rejecting those more viscid ones, which abound in the common mass from whence the selection is made. We witness, in short, in this system of vessels, the perpetual exertion of an influence which entertains a specific affinity for those fluids which it is their function to separate from a common material. It is to the exertion of this affinity in the capillary system that I am disposed to look for an auxiliary power to the heart, in the performance of the circulation." If the existence of this capillary attraction, or vital affinity, be admitted, we can be at no loss for a power sufficient to explain all the phenomena in question; and it must be acknowledged that a variety of circumstances are calculated to give it support. If a ligature be applied to an artery, the vessel will become empty on the side farthest from the heart; and this may be accounted for, either by the tonic contraction of the artery, or by the impetus which the blood had previously received carrying it onwards. But, if both these possible explanations be guarded against, it becomes difficult to admit of any other means of emptying the vessels, except that of attraction in the minute branches, as it were sucking in the blood. The following experiment has been proposed, as coming under this description:—A portion of elastic-gum or other tube is to be substituted for a like portion of artery, and the blood allowed to flow through it. A ligature is to be *strained* on the artery at the part which has the tube between it and the heart; while a ligature is to be tied on the portion of artery nearest the heart. The blood is to be retained a few seconds in the tube, that it may lose its impetus, and the ligature which was strained is to be loosened. The portion of the part is to be kept perpendicular, that the blood may not be assisted by the force of gravitation; and, after a short time, the ligature, which had been loosened, is to be tied, and the tube examined: if it does not contain blood, our author argues that "it appears a legitimate inference that some power of attraction in the distribution of the branches assists in conveying or deriving blood from the trunks." (p. 132.) Dr. Pring does not appear to have established the fact by any satisfactory experiments, having a "general aversion" to performing them on living animals. It cannot, however, be made matter of complaint against many physiologists of the present day, that they give way to their feelings in this respect; some, particularly among our continental neighbours, appearing to bear with the most praiseworthy stoicism the systematic course of torture which they inflict upon the subjects of their experiments. Indeed, when we reflect upon the entire want of method, and even of obvious design, in varying these *ad infinitum*, we cannot but feel convinced how much easier it is to multiply facts, than to draw just conclusions from them.

To return to the subject of determination.—It appears that the second cause assigned, viz. an accelerated action of the arteries themselves, is inadmissible; and we proceed to consider the agency of dilatation of the arteries of the part.

That the vascular system possesses an absolute power of dilatation, is extremely improbable, and at all events has not been demonstrated; our author, therefore, supposes it to be passive during this operation; "not that the vessels dilate, but that they are dilated:" but, as the blood is held to be equally passive, we must, according to this view, of necessity have recourse to some other agency. This agency is the one we have already been inquiring into, and is supposed to consist in the "derivative" power of the secerning system, which, being increased, gives rise, as a consequence, to local dilatation of the arteries.

The author next proceeds to discuss the two following propositions: 1st, that determination of blood exists in every disease; and, 2d, that it is the cause of every disease. With regard to these positions, no one, we believe, who has been in the habit of examining, after death, the part supposed during life to be the seat of disease, can for a moment entertain the slightest doubt. Dr. Pring gives two cases to prove that turgescence of vessels may not be found on post-mortem examination, even although the symptoms may have indicated determination of blood to a particular part. A girl, who had been subject for two years to fits connected with irregular menstruation, died, after having been convulsed and insensible during a night: no turgescence, nor other sign of increased vascularity in the brain, were observed. A man, about forty years of age, was affected with pain in the head, vertigo, &c. He was repeatedly bled by his attendant, and lost much blood by hemorrhage from the nose: in three months he died in convulsions. The brain was the palest our author ever saw. This case exactly resembles that of a man bled to death; and we repeat, that none accustomed to morbid dissection can require any experiment in refutation of this proposition; and if we deny that determination of blood is to be admitted as an universal accompaniment of disease, it becomes unnecessary to enter into the second question at all. It is further to be observed that, as the blood itself is passive, any undue distribution of it must be effected by the agents of such distribution, which is thus found to be an effect, and not a primary cause, of disease.

"But it is chiefly or wholly from the frequency of the connexion of an undue quantity of blood with local disease, that its universality as a cause of disease has been inferred. It is proved, by its being preceded by disease, that the presence of an undue quantity of blood is not necessary to this effect, or that it is not the primary cause of disease. And I apprehend that the priority of a preternatural quantity of blood to the other phenomena of disease, (which, to give it the import of a cause, it is necessary to establish,) cannot, in any case, be very readily distinguished.

"Appealing to the order of succession, the strictest scrutiny will not enable us to perceive that the determination of blood precedes the other phenomena with which it is associated. In inflammation, for example, a sense of uneasiness or of pain precedes the visible swelling or enlargement of the blood-vessels. This precedence may be distinguished by those who have suffered acute inflammation of the phlegmonous, or of perhaps any other kind. But if, in the cases of spontaneous inflammation, it should still be denied that other symptoms of the commencement of disease precede the enlargement of the blood-vessels, this precedence must be admitted where the order is, by a clear perception of the period when the action of the cause commenced, perfectly obvious to the senses. Thus, if inflammation supervene upon an external injury, the first effect of that injury (as a puncture, perhaps, under the nail,) is to produce exquisite pain; heat, throbbing, and tumefaction succeed: but the state of irritation, denoted by the pain first consequent upon the infliction of the injury, certainly precedes all the visible phenomena which ensue, and with the occurrence of which vascular dilatation is synchronous, and also co-operative with the other circumstances of a diseased state, in the whole series of effects which might succeed to the external injury.

"Thus it appears, agreeably with the strongest and most unexceptionable testimony, that the determination of blood to a part is a consequence of the assumption of a state of disease; that it is a part of a diseased state, which it helps to establish, and that it can be at most but auxiliary to the other causes involved in the condition of disease, in deciding its varieties and duration." (p. 153--4.)

After some further argument and illustration, our author comes to the following general conclusions:

"1. That a part cannot obtain a preternatural quantity of blood by the exertion of any power which belongs naturally to the arteries.

"2. That the ascertained powers of the arteries, viz. their tonic and elastic powers of contraction, may be overcome by the causes which produce determination of blood.

"3. That a preternatural determination of blood, although a general accompaniment, is not found to be an invariable one, of disease, in seats where the evidence of such determination has been looked for.

"4. That a determination of blood is occasioned by a local state of the structure, and cannot be produced by any action of the heart, which must be equally relative to the whole vascular system; or by any condition of this organ, except such as presents mechanical obstruction to the passage of the blood through its cavities.

"5. That the determination of blood does not commence disease, or is not the antecedent of this condition.

"6. That determination of blood is preceded by the assumption of a state of disease, which is denoted by symptoms; which state of antecedent disease, merely as it requires some term, may be expressed by the words irritation or excitement.

"7. That, as the recognised powers of the circulation are inadequate to account for this phenomenon, the concurrence of a function of the secerning system has been supposed necessary to this end.

"8. That the secerning system consists of the termination of arteries, which separate fluids from the blood by an affinity with these fluids, which is an exertion of the properties of life.

"9. That this function constitutes a power of attraction at the extremities of the arteries, which helps to carry the blood through its course, and is at once auxiliary to the heart in the circulation of the blood, and a centre of the power which makes nutrient fluids pervade the molecules of the structures; and is also capable of compelling their return through the absorbents into the system of the blood-vessels; although it is probable that the absorbent orifices have a similar function, which gives additional strength to the vis à tergo, by which fluids might be otherwise conveyed through them." (p. 163—165.)

[To be continued.]

---

ART. V.—*Prison Labour, &c.—Correspondence and Communications addressed to his Majesty's principal Secretary of State for the Home Department, concerning the Introduction of Tread-Mills into Prisons, with other Matters connected with the Subject of Prison Discipline.* By Sir JOHN COX HIPPISLEY, Bart. D.C.L. F.R. and A.S. a Bencher of the Inner Temple.—8vo. pp. 228. London: G. and W. Nicol, Pall-Mall. 1823.

THE subject of this work is of such paramount importance, in a public point of view, that we should consider ourselves as culpably negligent of our duty as journalists if we delayed, even for one month, to give as full an analysis of its contents as our limits will permit. The tendency of this book will, no doubt, very much startle and appal those who have considered the introduction of the tread-wheel in our prisons as the death-blow to crimes and misdemeanours of all descriptions and degrees, and who have expected from its employment a practical illustration of the doctrine of the perfectibility of human nature; yet we are much mistaken if a careful perusal of its contents will not do very much to check and counteract the current of popular opinion, which has hitherto flowed, almost without impediment or opposition, in favour of this powerful instrument of punishment. The public is indeed, we conceive, highly indebted to the distinguished author for the pains he has taken in the investigation of this subject: he has spared neither time, trouble, nor expence, in pursuing this inquiry; and he appears to have devoted himself to the task with that zeal for the public good which is so characteristic of the English gentleman, and which looks only for its reward in the consciousness of having performed a duty.

A popular writer, not much more than half a century ago, has put the following sentence* into the mouth of one of his most entertaining characters: "People may say this or that about being in jail, but, for my part, I found Newgate as agreeable a place as ever I was in all my life: I had my belly-full to eat and drink, and nothing to do;" and we have no doubt that this would be the opinion of a great majority of those whose principal objection is *to work*, and who would consider a successful course of roguery easily expiated by a few months' residence with companions of their own stamp, allowing them sufficient leisure to recruit their strength, and to devise more ingenious modes of following their lawless pursuits. This severe satire upon the state of our prisons was, we fear, but too just at the time it was written, and many years elapsed before any successful endeavours were made to alleviate or remove those obvious evils, notwithstanding the examples of Holland and America, where plans for the exercise and improvement of prisoners had for some time been in operation: indeed, as late as the year 1797, a writer in the Encyclopædia Britannica maintains that a prison is only a place of safe custody, and not of punishment. Other and more enlightened notions have, however, of late years, gradually made their way; the exertions of benevolent individuals, and the active operation of the Prison Discipline Society, have altogether changed the character of our places of confinement; and, in fact, they had latterly become such enviable residences, as to render some kind of labour and discipline absolutely necessary: the only contest, then, at present, is with respect to the *mode*. All are agreed as to the propriety of the general

* Goldsmith's Essays.

principle, of which the author of the work before us is one of the warmest advocates, and it is only against a particular system that he has lifted his voice; and the objections which he urges against the tread-wheel are not only strong, but in many points unanswerable; and that they are so, we are further led to infer from this circumstance, that the application of this mode of discipline is practically forbidden in so many instances, as to render it, even at present, any thing but a general instrument of punishment; for it is evident that, if women, if the ruptured, if the scrofulous, and those affected with varices to any extent, are considered unfit for the exercise of the wheel, a great proportion of prisoners must either be left idle, or some other mode of labour must be devised for them;—but we anticipate.

Before we introduce the work itself to our readers, we may just remark that the objections against the tread-wheel divide themselves into two branches,—political and medical. With the first *we* have little to do; but we may be allowed to hint, that, where height and strength make so great and obvious a difference in the quantity of suffering, the indiscriminate employment of the wheel cannot be just, unless it be clearly proved that physical strength and moral turpitude always bear a relative proportion to each other.

Having made these preliminary observations, we shall proceed at once to examine the volume; and, as we have already shown that our opinions are pretty much in unison with those of the author, we shall not have occasion often to interrupt the narrative with many remarks of our own. In pursuing our inquiry, we have ventured to deviate from the arrangement of the author, as the space we are enabled to devote to the subject obliges us to avoid repetition as much as possible, and to arrange our matter in the smallest possible compass. The work is divided into four principal parts, or sections, occupying 195 pages,—first, an Introduction; secondly, a copy of a letter to Mr. Secretary Peel, with the original statements transmitted to the several clerks of the peace, and in which is comprised the bulk of the medical evidence on the subject; thirdly, copies of all communications made to the Secretary of State for the Home Department, respecting tread-wheels in gaols or houses of correction;—and an Appendix, containing extracts from the evidence of Sir J. Acland and the author, before committees of the Houses of Lords and Commons, on the subject of prison discipline; and a note from Sir J. Hippisley to the Shepton Prison Committee, and which is of an interest comparatively local.

After some prefatory remarks upon the changes produced of late years in the condition of our prisons, our author mentions the introduction of the tread-wheel, under the auspices, and by the recommendation, of the committee of the Prison Discipline Society, and then adds—

"To the principle of hard labour, as fairly intended by the statute, so far from being an enemy, he is a most zealous friend: but, during a considerable portion of a long-protracted life, having been much occupied in the duties of the provincial magistracy in the counties of his usual residence; and having for many years, as a visiting justice, given an especial attention to the most considerable house of correction in the county of Somerset, he has viewed, with more than an ordinary interest, the extreme to which this re-action in the public feeling has led; and, particularly, the popularity it has given to the very expensive and enormous machinery of the tread-wheel: which he has found, from his own repeated investigations, and those of many enlightened and intelligent friends who have engaged in the same inquiry, to be highly mischievous in its principle, and baneful in its effects to those who are so indiscriminately sentenced to it; and, consequently, an instrument which neither the government nor the people of this country can countenance, when its evils are fully laid before them.

"It was under this impression he felt himself called upon to address the following communications to his Majesty's principal Secretary of State for the Home Department. That minister appears to have felt the importance of the object, by the motion he afterwards made in Parliament; nor does it seem, from the tenor of that motion, that he originally intended to abridge the limits of inquiry; but the subsequent official circular, addressed to the visiting magistrates, is so pointedly and locally restricted, as necessarily to have produced a result wholly inadequate to the purpose of such information as seems requisite to assist the judgment in determining the merits of a question that, in fact, ramifies into many important branches, though limited, in the first step, to the introduction of the tread-wheel. Any observation, however, upon the official communications contained in the return by order of Parliament, and printed on the 10th of March, will be reserved, till some reference be made to those anterior communications which have been excluded by the restrictions of government of the 18th of January. In the actual circumstances, the writer has thought it incumbent on him to submit the evidence entire to the public, as it will be found to develop and establish, from incontrovertible, and in most cases concurrent, testimony, a variety of weighty facts, which must necessarily be of interesting reference to the members of the legislature; many of whom, probably, at the present hour, are occupied with the provisions of the revived Gaol Bill in its progress through Parliament." (p. 4, 5.)

To Mr. Cubitt, of Ipswich, must be assigned the merit of having introduced the use of the tread-wheel in prisons, but the invention is of a much older date; and it appears to have been applied in this country as a crane at the warehouses of the East-India Company, having been introduced by a Mr. Hardie, who obtained a patent for the construction; though, in fact, he had no pretension to the discovery of the principle, as a wheel of this description had long been employed by the Chinese in the irrigation of their plantations. The description and engraving of the crane, as given by Dr. OLINTHUS GREGORY, leaves no doubt as to its identity with the tread-wheel. Our author, however, desirous of ascertaining the present state of the machinery in the East-India Company's warehouses, procured a report to be drawn up by the principal officers of the warehouse department, from which it appears that serious accidents have occurred in the use of these cranes; that they are considered more dangerous than the capstan; that in two instances labourers have been so severely hurt, as to be pensioned by the Company; and that, finally, the cranes were taken down last summer.

"If, in the selection of the quality or implements of prison labour the personal security of the individual subjected to the discipline be held to constitute a reasonable consideration, and to attach a just responsibility upon those who hold that selection, it should seem that, antecedently to the period when it was adopted by Mr. Cubitt, but little could be collected from the test of experience to warrant the introduction of the tread-wheel.

"With this conviction on the part of the writer, the result of the most deliberate and repeated personal examination and inquiry, in concurrence with professional characters whose opinions he held himself bound to respect, the original communication was first made to his Majesty's principal Secretary of State for the Home Department, and is now submitted to the public." (p. 10, 11.)

From that investigation, the following facts (which we have compressed) appear to be established: 1st. From the enormous height, complication, &c. of the tread-wheel, there is great danger of its breaking; and which has actually occurred four times within three months in the house of correction in Cold-bath Fields, whereby all the prisoners on the wheel must be thrown down on their back from a height, with a chance of considerable injury. 2dly. That the posture on this machine implies an exertion so exhausting to the human frame, as not to be enforced for more than a *quarter of an hour* at a time; whilst at Edinburgh only seven minutes and a half are ventured to be risked. 3dly. That, in consequence of the exhaustion produced by this exercise, a fuller and richer diet has been humanely allowed at several prisons, particularly at Edinburgh and North Allerton. 4th. That strains upon the organs and muscles immediately called into exercise, have taken place on various occasions. 5th. That, in consequence of these united causes, many of the female prisoners have been suddenly obliged to descend from the tread-mill under circumstances highly indelicate, and in consequence have been in most prisons exempted from this kind of labour. 6th. That a similar *kind of labour* has been found to produce rupture and varicose veins in other classes; and that, consequently, the same effects may be apprehended from this; and, therefore, 7th, that prisoners labouring under these complaints, or with a tendency to them, are exempted from the punishment of the tread-wheel. 8th. That, for these reasons, the unhappy culprits have a horror of this discipline, and would rather undergo any fatigue, or suffer any deprivation, than return to the house of correction, when once released; and that, 9th, notwithstanding its enormous expence in erecting, in consequence of the above reasons, it is of very limited application, and cannot be exercised over one half the delinquents. 10th. That in this mode of punishment there is only one degree as well as kind, so that criminals of every shade of character are subjected to the same *degree* of severe and perilous suffering; and, 11th, as a necessary consequence, that it is absolutely expedient, for the purposes of justice, as well as for the salutary application of hard prison labour, in the spirit as well as letter of the statute, that means of discipline very different from the tread-wheel should be resorted to. This leads our author immediately to the consideration of the Hand-crank Mill, of which he is a warm admirer and advocate; but we shall for the present pass over this part of his inquiry, and pursue our consideration of the valuable and interesting medical communications, which follow next in order, and tend most fully to confirm and verify the objections which we have quoted above. In this list we find the names of Dr. GOOD, Sir GILBERT BLANE, Mr. COPELAND, Mr. COLE, a gentleman whose name is not mentioned, Sir W. BLIZARD, Mr. HICKES, of Bath, Mr. DAY, of the Isle of Wight; and to these we may add the valuable and weighty authority of Dr. PARIS, in his last work on Medical Jurisprudence.* Of these gentlemen, Dr. Good seems to have considered the subject with the greatest attention, and his letter appears to us to embrace the whole question; for, although numbers and respectability give weight and strength undoubtedly to the opinions he has expressed, yet, as no fact or observation of importance has escaped his penetration, our quotations will be principally derived from that source, though we may, for obvious reasons, make our extracts not exactly in the order in which they occur in the letter itself.

After descanting at some length upon some discrepancies in the reports of the medical men from various prisons where the tread-wheel is in operation, Dr. Good thus describes *what he saw* in the house of correction in Cold-bath Fields:

"The wheels were at work on our arrival in all the yards, still idly expending their power, and that of their workers, *in the air*. The hour was half-past eleven in the morning, the thermometer at 60° Fahrenheit, with a cool and gusty breeze, which many have complained of as being

* Vol. iii. pages 151—3.

chilly, veering from north to south-west. We examined the subterranean machinery, which, with the ponderous fly above, was working at a fearfully rapid rate, notwithstanding the slow-paced motion of the principal shaft. The men were on duty on the wheels in their respective yards, and the report is true that the shaft has again broken, forming a fifth instance of failure; and other workers been again thrown upon their backs on the raised platform, and must in some instances have fallen through to the stone pavement, some ten or twelve feet below, had not the present vigilant governor, in anticipation of such an accident, prudently ordered the middle hatchways to be closed. I inspected the men as they descended in rotation from the wheel, at the end of the quarter of an hour's task-work, and made room for fresh relays. Every one of them was perspiring, some in a dripping sweat. On asking them separately, and at a distance from each other, where was the chief stress of labour, they stated in succession, and without the least variation, that they suffered great pain in the calf of the leg, and in the ham; whilst most of them, though not all, complained of distress also in the instep. On examining the bottom of their shoes, it was manifest that the line of tread had not extended farther than from the extremity of the toes to about one-third of the bottom of the foot; for in several instances the shoes were new, and between this line and the heel altogether unsoiled: a fact, however, that was as obvious from the position of the foot while at work, as from the appearance of the shoe at rest. Several of the workers seemed to aim at supporting their weight by bringing the heel into action, the feet being twisted outwards; and, on inquiring why this was not oftener accomplished, the reply was, that, though they could gain a little in this way, it was with so painful a stress of the knees, that they could only try at it occasionally. The palms of their hands, in consequence of holding tight to the rail, were in every instance hardened, in many horny, in some blistered and discharging water. The keeper, who accompanied us, admitted the truth of all these statements, and added, that it was the ordinary result of the labour! and that use did not seem to render it less severe: for those who had been confined long appeared to suffer nearly, or altogether, as much as those who were new to the work; thus confirming a remark I long since took the liberty of making to you: I mean that, when an organ is directed to any kind of labour for which it is not naturally intended, no perseverance will ever give it facility of action, or take off the original distress." (p. 31—33.)

Dr. Good then proceeds to examine *seriatim* the evils to be apprehended from this mode of labour; and, first, with respect to the production of premature and excessive menstruation. It appears that, out of twenty-four prisons, women are only subjected to the tread-wheel in four,—viz. Exeter, Dorchester, Cold-bath Fields, and Brixton. The words of the reporter relative to the prison at Exeter are at least equivocal; from Dorchester, there is at once an admission that certain complaints have occurred, and which the surgeon has attributed to working at the wheel, although they had only had five months'

trial of that discipline; the same result was also acknowledged in Cold-bath Fields; whilst from Brixton we are informed that the tread-wheel is an excellent remedy for rheumatism, and a preventive against weaknesses and varicose tumors. In this place we shall go back a few pages, in order to point out the assertion of the governor of the Bedford house of correction, who says that the labour *is not severe;* whilst the surgeon to another institution of the same kind is indiscreet enough to affirm, that, after a few days' work on the tread-mill, the employment *ceases to be a punishment,*—thus ingeniously contriving to give the death-blow to the invention he intended to eulogize.

"I have dwelt more at large upon this subject, (continues Dr. Good,) because the principle upon which it hinges is just as applicable to males as to females, and forms the basis of by far the greater number of the complaints anticipated on first contemplating the discipline of the tread-wheel. For, if the muscles and organs of the loins and lower part of the body be urged to excess, and pressed into an unnatural and distressing, and hence into a morbid, play upon each other in the case of the latter, so must it also be in the case of the former. The greater firmness, indeed, of the male structure must necessarily resist its evil effects for a longer period of time, in consequence of which they will neither so soon nor so frequently show themselves: insomuch that, as I have already observed, it may in some instances require several years before the natural strength of the organs will decidedly fall a prey in the contest. But the battle is still waging, though unperceived; the tread-wheel is still gaining ground; and, not only upon the field of combat, but even afterwards, when released from it, the stoutest champion in this new system of warfare may for the first time give palpable marks of its mischievous effects." (p. 38, 39.)

Continuing the examination of the Reports, we find, in the first, a rupture produced upon a prisoner labouring on the wheel. The last, from Edinburgh, contains a case of spitting of blood and a bruised ankle; at Lancaster, an inflammation and tumor in the groin; and Mr. Green, writing from Durham, where the mill had only been employed eight or nine months, says, "I am of opinion, if persevered in *with prudence, and not too long continued,* no serious effects are to be apprehended from its use: and, in short, the various modifications proposed by different practitioners in the position of the hand-rail, and in the intervals between the steps, sufficiently prove they have respectively thought there was a necessity for some kind of alteration; and which necessity could only arise from the uneasiness and undue exertion produced by the position of the body and the mode of labour.

"Nothing, indeed, can more decisively prove the distress and undue exertion under which the muscles chiefly pressed upon labour, than the extreme and exhausting perspiration into which, during warm weather, the prisoners are thrown within a few minutes, and which the mere quantity of labour is altogether incapable of accounting for. This is one of those evils already enumerated as having existed, last October, in the house of correction in Cold-bath Fields; and which is also glanced at in the government document by Mr. Tucker, in his Report from Exeter. 'I learned only (says he,) that the muscles of the legs sometimes ached, and that work on the wheel in warm weather would produce a great perspiration.' The same fact is admitted by Mr. Dent, to whose active and praise-worthy exertions the North Riding of Yorkshire is under great obligations. 'I admit (says he,) that the employment may cause men to perspire, and, unless means are taken to ensure the freest respiration, perhaps profusely. At first we found a tendency to the inconvenience complained of, but it was completely obviated by substituting an open trellis instead of closed boards.' This, however, by no means always answers, though it shows another necessity for some modification in the general system of the tread-wheel discipline, according to the ingenuity of the contrivers. In the Cold-bath Fields prison, the men work under sheds in the open air; and yet here the perspiration, at the time referred to, was not only profuse, but highly exhausting. Nevertheless, I do not urge this as an objection *in se*, nor have I ever thus urged it: for, like yourself, I am altogether friendly to hard labour as such, and care but little how hard it may be, provided the health of the prisoner is not hereby put in jeopardy. But I mention the fact as a strong and incontrovertible proof of the trying and distressful nature of the present labour, however hard or mitigated; or, in other words, as painful, and therefore morbid, from its quality, and not from its quantity. With respect to the latter, I entirely agree with Mr. Dent, and would even go beyond him, that 'the voluntary efforts of honest industry are surely not too high a measure for the standard of compulsory labour: and where is the labourer whose daily task does not exceed a walk of two miles, even admitting it to be up-hill?—yet this is as great a length of distance as can be performed by the revolution of our tread-wheel in six hours, the average of each man's labour at it per day.' I now, however, take this distinguished and excellent magistrate upon his own ground, and ask him, in reply, where is the hill, with a path already cut up it, in which any man in a state of health would be thrown into the slightest perspiration in ten or fifteen minutes, even admitting it were covered over, (as in the case of stairs in a house,) whose pace should only equal that which is here calculated, being not more than one-quarter of a mile in three-quarters of an hour? Who does not see in this, as well as in every other result we have already contemplated, that the alleged cause and effect are not commensurate, and consequently that there must be some other, and more morbid, power than that of mere progression? Who does not at once dive into the real source of that secret and indescribable horror which this labour is universally allowed to excite, and which has never been satisfactorily accounted for otherwise? And who, at the same time, does not enter into the absolute necessity, and admit in its fullest extent, the wisdom of those numerous emancipations from labour which exist in almost every prison into which the tread-wheel is at this moment introduced, and which renders it only available to considerably less than half those for whom it was at first designed? That it is totally unfit for women, and will in a short time be universally abandoned in respect to them, does not now, I believe, admit of the shadow of a doubt. Yet, among males, the ruptured are, I apprehend, as universally exempted, amounting, according to the estimate of Mr. Macelwain, surgeon to that truly valuable association, 'the London Truss Society for gratuitously relieving the poor that are afflicted with ruptures,' to be not less than one in six of the labouring classes, as nearly as can be calculated. To which we are to add the consumptive, who are humanely spared, as noticed, in the Cold-bath Fields and various other prisons; and those labouring under venereal complaints, scrofula, or diseases of any kind in the groin, all of whom it is judged necessary to exempt at Lancaster Castle." (p. 42—46.)

At Lancaster Castle, it appears that, at the date of the surgeon's report in February, the prisoners on the wheel had gained weight; but, by carrying the inquiry down to the month of May, a very different result was produced. In most prisons, the time devoted to actual labour is seven hours and twenty minutes, during which period the prisoner walks about two miles and one furlong; whereas at Lancaster the standard has been only seven hours, so that the prisoners have only walked two miles a-day, and, in short and cold days, one and a half miles. It appears that, as long as this rate is continued, the scale of diet being exactly similar to that recommended by the visiting physician at the Penitentiary at Millbank, that the workers have increased in weight from eight or nine grains to one ounce and a half in the day; but, if the labour is pushed to two miles in the day, the prisoners waste at the rate of from a pound to nearly a pound and a half in three weeks.

The next point which Dr. Good proceeds to examine, is the probability of the production of herniæ, and aneurismal or varicose tumors in the vessels of the lower limbs, by this species of labour; but, as these are diseases of slow growth, the fact cannot possibly be proved otherwise than by a reference to the condition of those classes of men whose mode of labour bears the greatest analogy to that of the tread-wheel. From the testimonies which he has produced in support of this opinion, as far as relates to sailors and miners, we conceive that he has established his point to the satisfaction of the most sceptical; though, for our own parts, the tendency of this kind of labour is so obvious, that we scarcely think so great a quantity of proof necessary to establish so self-evident a proposition. We cannot, however, forbear quoting a passage from another letter of Dr. Good's on the same subject, and which occurs at p. 133:

"Varices and aneurisms, or tumors of the veins and arteries, may arise in any part of the body; but they occur most frequently in the lower limbs, from their dependent condition; and they never can be cured, or even prevented, where there is a tendency to the disease, but by counteracting this dependency,—as by rest, a reclined position, and the uniform support of a bandage, or laced stocking. Upon this subject there never has been, and never can be, any dispute in the profession, as must be obvious from the nature of the case. This may be general or local: it may proceed from an uniform weakness in the tunics of the arteries or veins; or from weakness in particular parts, produced by cramp, strain, or stretch in such parts, and hence, very generally, by violent or fatiguing exertion, or standing long on the legs. Hence hardfaring men, and especially hardfaring women, are particularly liable to these complaints; as are officers in the army, when exposed to great harassment and exhaustion; so are pregnant women, from obstructed circulation. Now a man would be laughed at who should recommend the tread-mill, or any thing like it, as a cure, or a mean of prevention, where a tendency exists, to any of these patients; and to talk of promoting or increasing the circulation by any means where the vessels are too weak in particular parts to bear its ordinary force without bursting, is most extraordinary."

Sir W. Blizard expresses himself thus:

"The communications No. 1 and 2 contain also, principally, my own anatomical and physiological ideas of the comparative effects of the two species of labour. I will, however, add one or two other remarks. On the tread-machine, the abdominal muscles are kept in constant action, and the extensor muscles of the back always on a stretch. Now, for free respiration, the abdominal muscles and diaphragm should act alternately. Free respiration is necessary for the due aeration of the blood; and a proper aeration of the blood, by the vessels of the lungs, is required for the proper condition of the blood, with reference to every important function of the body, and forms the most powerful preventive of typhous fever. For the foregoing reasons, the exercise should be as much as possible in the open air.

"The preference expressed in the extracts* is founded well on the consideration of fitting the muscular power of the arms to future labour, and hence the more ready performance of duties for subsistence, and a preventive of inducement to criminal means of obtaining it." (p. 105.)

—and we might go on extracting passages from other authorities to the same effect, almost *ad infinitum;* but those which we have selected cannot fail to produce a proper effect on the mind of the unprejudiced reader.

It will, however, be naturally asked, what is the nature and extent of the evidence on the other side of this question? and what is the tenor of the Reports from the various prisons in which the tread-wheel is employed? We find, in the work before us, reports from twenty counties, in most of which the wheel had only been employed for the space of a few months: yet, out of these twenty reports, we have already noticed that

* In favour of the hand-crank machinery.

opinions *unfavourable* to the *general* employment of this discipline are expressed by six, at least, of the surgeons; that, whilst one* is for excluding those with ruptures or syphilis from its exercise, a second† recommends it to be used with caution; a third‡ reports a rupture to have been produced by a violent fit of coughing on the wheel; a fourth§ relates a case of spitting of blood, which also took place whilst undergoing the same discipline, but which, as he heard no more of the case, the reporter attributes to a desire to escape from the labour. One or two other minor accidents are also related, but which we will not insist upon: neither will we stop to point out the inconsistencies displayed by one or two of the warmest advocates of this potent machine; because we think that, considering the short time it had been in use in most of the above instances,—considering the prejudice that was existing in its favour at the time, and the great moral benefits that were expected from it,—we are only astonished that so many objections forced themselves upon the notice of those who made the reports.

So far we conceive we have placed in a prominent point of view that part of our author's work in which he urges his objections against the tread-wheel; and we now come to consider the plan which he recommends as a substitute for it, and of which we have purposely forborne to speak hitherto, although in the work itself the comparative merits of the two are frequently contrasted with each other; and much of the medical evidence given expresses a decided conviction of the superiority of the hand-crank machinery. Reverting, then, to page 16, we find the following reasons for giving a preference to this latter mode of labour:—That it affords the workers the natural position of standing firm on the feet, and on firm ground; that it divides the exercise equally among those organs intended for muscular motion; increases the general health and strength, instead of counteracting them; and *hereby prepares every prisoner, so worked, for applying himself with greater facility to a variety of handicraft and other trades, after his discharge from confinement, than he possessed before his commitment to prison;* and renders, in fact, the habitual use of hard manual labour a great and permanent good, instead of what may possibly be a serious and lasting evil. This last parapraph we conceive to be of the highest importance, and of itself quite decisive of the question. But it has been urged that the operation of the tread-wheel is so effectual in the prevention of crime, that in those prisons where it is employed the average of recommitals is reduced almost to nothing; but, by examining the third Report

* Lancaster Report. † Durham ditto.

‡ Berkshire ditto. § Edinburgh ditto.

of the Committee of Prison Discipline, we shall find that, in those prisons in which the hand-crank machinery was in use, this average was even lower, as the following statement will show:—Preston, four per cent.; Wakefield, four per cent.; Bury, five per cent.; Devizes, the general average three per cent.; Ipswich, three per cent.; Lewes, six per cent.; Worcester, two per cent.; Leicester, three per cent. Upon which Sir John Hippisley remarks, "It is not less fair to observe that, in the prison of Bury *alone* was the tread-mill *at that time erected*; and that at the Devizes, and at Worcester, the hand-cranks were in action; yet even Bury does not take the lead of the enumerated prisons for a *relative paucity* of recommitals." (p. 118.)

We have now only to confirm the above statement by quoting the opinions of the magisterial and medical authorities adduced by our author; and, in conclusion, to give as accurate a description as we are able, without the assistance of the engraving, of the improved hand-crank mill, as recommended by Sir John. In order to avoid needless repetitions, we shall only insert the two following extracts from Mr. COPELAND's and Mr. MACELWAIN's letters to Dr. Good:

"With respect to the general causes of hernia and of varices, two very common and very important diseases, I have nothing to remark more than is commonly known to the profession: but I should think those diseases much more likely to be produced by the efforts of labour of the tread-mill, than by the double labour of hand and leg, as sketched and described under the name of the hand-crank mill in the printed paper; which appears to unite the advantages of healthy exercise with those of compelled labour as a punishment."

"With reference to the different modes of labour, certainly those which call alternately into action different sets of muscles are to be considered as most contributing to the health and strength of the individual employed. And it would appear to me that the hand-crank mill is calculated, to a great extent, to meet the object, when employed in the manner you propose."

"There is, however, another ground, and of a still more important nature than any we have yet contemplated, which induces me to prefer the hand-crank mill to the tread-wheel. While the latter is purposely designed to operate by terror, and hence necessarily excites in the prisoner a dread and disgust of labour, and of all muscular exertion whatever, by which he becomes habitually unfitted for work of every kind upon his discharge from confinement, the former operates by giving an insensible invigoration and facility of action to the muscles of most importance in all the callings of mechanical and handicraft industry; and, consequently, habitually prepares him for providing for himself at the same period. We have here a moral attribute, to which the rival labour can make no pretensions. The culprit, just freed from the tread-wheel, though he should have escaped the diseases and injuries to which he has been exposed while under its domination, has gained nothing to facilitate his progress in any useful employment; with a greater hatred of a prison-life, he will have no greater means, and may perhaps have fewer, of avoiding it: while the hand-crank man will find that, under your improved machinery and regulations, he has been serving a most valuable apprenticeship, and has become initiated in the healthful and vigorous arts of thrusting, pulling, heaving, and bearing burdens; for the action of the cranks on the several muscular positions of the body in effect prepares it for the various relative details of manual labour. What was irksome to him before he went to prison, he now undergoes with ease, and even relish: his punishment may have been severe, but it has proved wholesome; and the curse of earning his miserable pittance of bread and water with the daily and profuse sweat of his brow, is transformed into a blessing for which he will have to thank the magistracy, and the manual labour of the crank-mill as long as he lives." (p. 64–66.)

We conclude our Extracts with giving the opinion of Sir J. Acland, one of the oldest and most active magistrates of the county of Somerset.

"The hand-mill, I agree with you, is free from all such complaints, and the labour of it may be increased to any quantum of power necessary to produce hard labour, or be regulated to any degree of wholesome exercise. Can any one who will exercise his reason, by the help of a little anatomical knowledge, halt between the two opinions. The first dangerous result from the tread-mill will open the eyes of the blind and unthinking."

We have thus endeavoured to give our readers a sketch of Sir J. Hippisley's work, and in doing so we have been more attentive to placing the facts he has collected in a strong point of view, than in following the exact order and arrangement which he has adopted: aware of the imperfection of this article, (arising principally, we hope, from the limited space we have been under the necessity of devoting to the subject,) still we have done enough, we presume, to call the attention of the public, and of professional men especially, to the subject. We shall be the means of disseminating widely the objections which appear to us to be urged in the spirit of justice and mercy, against this very unequal and unprofitable species of labour; and in so doing we shall have the consolation of feeling that we have acquitted ourselves of a public duty, and have, to the best of our abilities, furthered the benevolent design of the author.

We add the following description of an improved *hand-crank mill.* This consists of a central shaft, round which any number of cranks may be placed, so as to communicate the joint efforts of all the men to it at the same time; and they may be so partitioned as to preserve the classification of the persons at work. The position of the men may be varied at pleasure; a man may

[illegible] down the tread-wheels; put up the crank-[illegible]

be placed with his face towards the others, and the whole may be thus disposed alternately, by which the labour may be occasionally increased or diminished. The cranks should be covered with loose ferrils of plate-iron, as a protection for the hands; each ferril of a due length for the labour of a single man. Each crank carries a *bevil cog-wheel* at its extreme end, and all these wheels take in common into the *cogs of a horizontal bevil wheel*, bearing upon the perpendicular shaft, which passes through the ceiling, and gives motion to mill-work for grinding corn, or any other purpose; and to which the workmen have no access. Any one of the cranks may, however, be thrown out of gear at pleasure, by which the work will be rendered more severe upon those that remain. One of the cranks is also made adjustable, by means of *screw-nuts*, so that its *throw*, or the circle which it causes the hands to describe, may be varied in diameter, without any sensible variation in the speed of the machinery. The room, which it would be desirable should be open to the air, in which the men work, should contain a clock, not only for the regulation of such mill-speed, but to determine the length of time during which each set of men is to work; there is also, at the other extremity of the room, a *counter*, or *tell-tale*, which, by means of a dial-plate, shows the number of turns that have been given to the machinery in the absence of the inspector, during any period of time.

---

### DIVISION II.

### FOREIGN.

Art. VI.—*A Treatise on the Materia Medica and Therapeutics.* By John Eberle, M.D. Editor of the American Medical Recorder; Member of the American Philosophical Society, of the Academy of Natural Sciences of Philadelphia; Corresponding Member of the Medico-Chirurgical Society of Berlin, &c. &c. In two Volumes.—Philadelphia: James Webster. 1822.

Nothing tends more clearly to illustrate the improvements which the healing art has undergone within the last half century, than comparing the histories of the materia medica of that period with those of the present. It was by no means uncommon to find fifty, or an hundred, or even more, ingredients blended together in one prescription, by which means it became impossible to ascertain, either the properties of any of the medicines taken individually, or to separate the efficacious from such as were inert or hurtful. If it must be allowed that we still have sometimes occasion to see medicines somewhat unskilfully* or capriciously combined, at least we have discontinued the exhi-

* The writer had occasion, at no very distant period, to hear of a few drops of sulphuric acid being added to a chalk mixture, in a case of diarrhœa.

bition of millepedes, "dead men's bones," and the farrago of disgusting remedies formerly held in repute: even the royal touch and the touch of a felon are now regarded as pretty much the same. In this country, the works of DUNCAN, THOMPSON, and PARIS, have greatly contributed to this good effect; and two recent American publications show that our trans-Atlantic brethern are not behind hand in this respect. The works to which we allude are Dr. CHAPMAN's and the one at the head of this article: it is of this latter we now proceed to give a short account.

The arrangement adopted by the author is that proposed by Dr. GRANVILLE, in the Number of this Journal for April 1822, which he is of opinion combines in some degree the advantages both of CULLEN's system and that of ALIBERT. To this choice we have no objection, conceiving it of more importance that a good account of the various medicines in use should be given, than absolute accuracy should be observed in their arrangement. Some great general divisions, indeed, are requisite to assist the memory, and facilitate the acquisition of knowledge; but we believe any of those in use are sufficient for all practical purposes. There is in the human mind a natural tendency to classification; and when we have the means of founding this upon obvious and undeniable relations, the advantages resulting to science are of the highest importance: witness the various branches of natural history, botany, mineralogy, and zoology. Here the arrangements are founded, not on occult or supposed properties, but on external and visible characters. Not so in medical science: here we have to trace the relations borne by external agents to living bodies,—relations so complicated and obscure, as to account most satisfactorily for the imperfections of all the systems of materia medica hitherto proposed, and leave us but little hope of seeing this defect very speedily removed.

The words of a recent French writer seem applicable to this subject: "Why reverse the natural order of things, the progressive march of knowledge? To create the classification before the facts, is it not, to use the expression of a celebrated author, *vouloir arranger une chambre vuide?* Is it not one of the weaknesses of human nature to wish to arrive at general conclusions before we have collected the details? Have the brilliant efforts of genius advanced science so much as the continued labours of a small number of individuals, born for observation, enlightened, studious, and modest? Let us cease, then, to attach to these systems, which are more or less arbitrary, a degree of importance which is much more imperiously required for the investigation of facts, and the search after truth. Nature derides our classifications and systems, and, while we vainly attempt to subject her phenomena to an arrangement purely arbitrary, she amuses herself with creating endless anomalies, which overthrow our systems, confound our theories, and seem to warn us that we are not permitted to raise the veil with which she conceals her sublime operations."*

The first chapter is devoted to a consideration of the *modus operandi* of medicines, chiefly with a view of invalidating the assertion made by Dr. Chapman, that "the ancient notion, which would refer the operation of medicines to their entrance into the circulation, is perfectly gratuitous, originating at a period of darkness, and when medicine was comparatively in its infancy; and is now abandoned by every one whose intelligence has at all kept pace with the progress of our science." We agree with our author, that this assertion on the part of Dr. Chapman is "entirely gratuitous." Opposed to it we have the testimonies of MAYER,† Sir E. HOME,‡ MAGENDIE,§ TIEDEMAN, and GMELIN;‖ and, should Dr. Chapman disregard the authority of foreigners, we refer him to the statements of his countrymen, Drs. HASLAN, COATES, LAWRENCE, MACNEVEN, ANDERSON, and DUCACHET; an account of most of whose experiments, taken from American Journals, has already been laid before our readers in former Numbers.

After this introductory chapter, Dr. Eberle proceeds at once to the consideration of particular classes of remedies, beginning with those which excite discharges from the alimentary canal. As a specimen of the author's manner, we select the description of two emetics, which we believe are little known in this country.

"*Lobelia Inflata.*—This is a biennial plant, and indigenous to the United States, in many parts of which it grows in great abundance. For a particular description of this species of lobelia, the reader is referred to Dr. Bigelow's 'American Medical Botany,' and to Dr. Barton's 'Vegetable Materia Medica,' of the United States, where accurate figures are given of the plant. The leaves and capsules of this plant are exceedingly acrid, and, when 'held in the mouth for some time, they produce giddiness and pain in the head, with a trembling agitation of the whole body; at length they bring on extreme nausea and vomiting. The taste resembles that of tartar emetic.'

"As an emetic, the lobelia inflata is extremely active, producing, in strong doses, 'great relaxation, debility, and perspiration.' Concomitant with its emetic operation, it sometimes acts on the bowels and produces purging. Shoepf merely notices this plant as 'astringent, and

---

* *Mémoire sur cette Question proposée par la Société de Médecine de Paris: "Determiner si, dans l'état actuel de nos connaissances, on peut établir une classification reguliere de Medicamens, fondée sur leurs propriétés medicinales."* Par M. CASS, Pharmacien, à Lyons.

† *Archiv für die Physiologie,* von J. F. MECKEL.

‡ *Philosophical Transactions,* 1811.

§ *Précis Elementaire de Phisiologie.*

‖ *Versuche über die Wege, auf Welchen Substanzen aus dem Magen und Darmcanal ins blut Gelangen, u. s. w.* Von F. TIEDEMAN, M.D. und L. GMELIN, M.D.

used in ophthalmia,' without saying any thing further concerning its medicinal powers. Our knowledge of its virtues as a medicine has been chiefly derived from the account given of its effects by the Rev. Dr. M. Cutler, whose experience with it appears to have been very considerable. He has found it particularly serviceable in asthmas. 'It has been my misfortune (says he,) to be an asthmatic for about ten years. I have made trial of a great variety of the usual remedies, with very little benefit. In several paroxysms I had found immediate relief, more frequently than from any thing else, from the skrunk-cabbage, (pothos fœtidum.) The last summer I had the severest attack I ever experienced. It commenced early in August, and continued about eight weeks. Dr. Drury, of Marblehead, also an asthmatic, had made use of a tincture of the Indian tobacco, (lobelia inflata,) in a severe paroxysm, early in the spring. It gave him immediate relief, and he has been entirely free from the complaint from that time. I had a tincture made of the fresh plant, and took care to have the spirit fully saturated, which I think is important. In a paroxysm, which perhaps was as severe as I ever experienced, the difficulty of breathing extreme, and after it had continued for a considerable time I took a table-spoonful. In three or four minutes my breathing was as free as it ever was. In ten minutes I took another spoonful, which occasioned sickness. After ten minutes I took the third, which produced sensible effects on the stomach, and a very little moderate puking, and a kind of prickly sensation through the whole system, even to the extremities of the fingers and toes. Since that time I have enjoyed as good health as, perhaps, before the first attack.' Other practitioners have employed this article with decided advantage in asthmatic affections. Dr. W. P. C. Barton mentions a case of this kind, in which he exhibited a tea-spoonful of the tincture every two hours, with speedy and obvious benefit. Dr. Stewart also, as I am informed by Dr. Barton, has obtained unequivocal advantages from the employment of this remedy in a case of spasmodic asthma. Dr. Cutler has found it a very useful pectoral 'in consumptive and other coughs depending on mucus accumulated in the bronchial vessels.'

" From my own experience I have nothing to add with regard to the remedial powers of this species of lobelia, in asthmatic affections. As an emetic, however, I have employed it in one case of croup, with very great benefit. I have also used it effectually instead of tobacco, in the form of an enema, to facilitate the return of a strangulated hernia.

" The plant should be collected in August, and plucked up by the roots. Every part of the plant possesses active qualities, but the roots and the inflated capsules are decidedly the most powerful. It may be given either in the form of powder or tincture: the latter is, however, the most convenient mode of exhibiting this remedy. The formula adopted in the American Pharmacopœia for making the tinctura lobeliæ, is given below.* As an emetic, we may give from ten to twenty grains of the powdered leaves, to an adult. In smaller doses its effects are expectorant. The saturated tincture is administered in the dose of from twenty to forty drops, to children of one or two years old.

---

* ℞. Lobeliæ uncias duas.

Alcoholis diluti octarium unum.

Digere per dies decem, et per chartam cola.

"*Spiræa Trifoliata.*—This is an icosandrous plant, and found in considerable abundance throughout almost every part of the United States. Its root is perennial, sending up annually several slender stems to the height of several feet, branching above, and of a reddish colour, It flowers in June and July, and delights in hilly woods or on the borders of rivulets. The root, which consists of 'long, brown, slender branches, radicating from a thick tuber,' is the part employed for medicinal purposes. It is said that the cortical part of the root alone possesses active qualities. 'The predominant soluble ingredients in this root appear to be a bitter extractive matter and resin. When boiled in water, it imparts to it a beautiful deep red-wine colour, and an intensely bitter taste. This decoction undergoes no change from alcohol or gelatine, though it gives a precipitate with muriate of tin. Water distilled from the root has its peculiar flavour, with little of the bitterness. A large portion of resin is precipitated on the addition of water to an alcoholic tincture of the root. From my own experience with this plant, which has not been inconsiderable, I am led to regard it as very little inferior to the officinal ipecacuanha, as an emetic. Like this latter article, it is a safe and efficacious vomit. While practising in Lancaster county, I employed this plant very frequently as an emetic, in the treatment of intermittent and bilious fevers, and it very seldom disappointed me of the desired effect. Dr. Bigelow however observes, that, from his own experience with this remedy, he was led to regard it as an emetic of very uncertain operation.

"Given with opium, I have found it particularly serviceable as a sudorific in dysenteric affections; and, from what I have observed of its effects in other cases, it appears to me that the opinion entertained by the late Dr. Barton of its possessing tonic properties, is not without foundation. In small doses, from two to four grains, I have taken it myself, when suffering from dyspepsia, and generally with evident advantage.

"As an emetic, it should be given in the dose of about thirty grains of the powdered root. It should be collected in September. Accurate figures of this plant are given in the first volume of Barton's 'Vegetable Materia Medica' of the United States; and in the third volume of Bigelow's 'American Medical Botany.'" (p. 63—68.)

We have looked over a considerable number of the articles, and can safely recommend them to our readers as in general very satisfactory, giving a good digest of the various opinions of practical writers respecting the use of particular medicines, and being highly creditable to the industry and judgment of the author. Before concluding, we would remark, that the labours of our trans-Atlantic brethren are less known and appreciated than they ought to be: the day is long past when the Americans were a set of mere traders and mechanics, occupied in providing for the wants common to infant colonies. Science is no longer confined to a few individuals, as a Rush or a Franklin; but a spirit of honourable emulation in the pursuit of knowledge is abroad among them, and tends, among other proofs, to mark them as a great and rising nation.

